# CIDR: Ultrafast and accurate clustering through imputation for single-cell RNA-seq data

**DOI:** 10.1186/s13059-017-1188-0

**Published:** 2017-03-28

**Authors:** Peijie Lin, Michael Troup, Joshua W. K. Ho

**Affiliations:** 1 Victor Chang Cardiac Research Institute, Darlinghurst, 2010 NSW Australia; 2 St Vincent’s Clinical School, University of New South Wales, Darlinghurst, 2010 NSW Australia

**Keywords:** Single-cell, scRNA-seq, Dropout, Imputation, Dimensionality reduction, Clustering, Cell type

## Abstract

Most existing dimensionality reduction and clustering packages for single-cell RNA-seq (scRNA-seq) data deal with dropouts by heavy modeling and computational machinery. Here, we introduce CIDR (Clustering through Imputation and Dimensionality Reduction), an ultrafast algorithm that uses a novel yet very simple implicit imputation approach to alleviate the impact of dropouts in scRNA-seq data in a principled manner. Using a range of simulated and real data, we show that CIDR improves the standard principal component analysis and outperforms the state-of-the-art methods, namely t-SNE, ZIFA, and RaceID, in terms of clustering accuracy. CIDR typically completes within seconds when processing a data set of hundreds of cells and minutes for a data set of thousands of cells. CIDR can be downloaded at https://github.com/VCCRI/CIDR.

**Electronic supplementary material:**

The online version of this article (doi:10.1186/s13059-017-1188-0) contains supplementary material, which is available to authorized users.

## Background

Single-cell RNA sequencing (scRNA-seq) enables researchers to study heterogeneity between individual cells and define cell types from a transcriptomic perspective. One prominent problem in scRNA-seq data analysis is the prevalence of dropouts, caused by failures in amplification during the reverse-transcription step in the RNA-seq experiment. The prevalence of dropouts manifests as an excess of zeros and near zero counts in the data set, which has been shown to create difficulties in scRNA-seq data analysis [1, 2].

Several packages have recently been developed for the various aspects of scRNA-seq data analysis, including cell cycle (cyclone [3] and scLVM [4]), normalization (scran [5]), differential expression analysis (scde [2] and MAST [6]), and temporal analysis (Monocle [7]), but few perform preprocessing steps such as dimensionality reduction and clustering, which are critical steps for studying cell-type heterogeneity.

The state-of-the-art dimensionality-reduction package for scRNA-seq data is ZIFA [1]. It implements a modified probabilistic principal component analysis (PCA) method that incorporates a zero inflated model to account for dropout events. ZIFA uses an iterative expectation-maximization algorithm for inference, which makes it computationally intensive for large scRNA-seq data sets.

Another package t-SNE [8] is popular among biologists, but it is not designed specifically for scRNA-seq data and does not address the issue of dropouts. Other recently developed tools, such as BackSPIN [9], pcaReduce [10], SC3 [11], SNN-Cliq [12], RaceID [13], and BISCUIT [14], were designed to deal with optimal clustering of single cells into meaningful groups or hierarchies. Like ZIFA, these algorithms usually involve statistical modeling, which requires estimates of parameters. These algorithms often make use of iterative methods to achieve local or global optimal solutions, and hence they can be slow when processing large data sets of more than several hundred single cells.

In many practical situations, researchers are interested in fast and intuitive clustering results that they can easily visualize. PCA is a common analytical approach for data visualization for sample heterogeneity, and is often used for dimensionality reduction prior to clustering. Many versions of PCA, such as the implementation prcomp in R, are very fast and have routinely been used for analyzing large gene expression data sets. Nonetheless, standard PCA is not designed to take into account dropouts in scRNA-seq data. In this work, we aim to develop a fast PCA-like algorithm that takes dropouts into account.

## Results

### Motivation

We note that PCA is equivalent to performing a principal coordinate analysis (PCoA) on an Euclidean distance matrix derived from the data set. We posit that as long as we can reliably estimate the dissimilarity between every pair of samples (i.e., single cells) in the presence of dropouts, there is no need to estimate explicitly the values of the dropouts.

Let us begin by examining the squared Euclidean distance between the expression profiles of two single cells, *C*_*i*_=(*o*_1*i*_,*o*_2*i*_,…,*o*_*ni*_) and *C*_*j*_=(*o*_1*j*_,*o*_2*j*_,…,*o*_*nj*_), where *o*_*ki*_ and *o*_*kj*_ represent the gene expression values of gene *k* in cells *C*_*i*_ and *C*_*j*_, respectively:

1 $$ \begin{aligned} \left[D\left(C_{i},C_{j}\right)\right]^{2}&=\sum_{k=1}^{n}{\left(o_{ki}-o_{kj}\right)^{2}} \\ &=\sum_{k\in\{\text{No zeros}\}}{\left(o_{ki}-o_{kj}\right)^{2}}\\ &\quad+ \sum_{k\in\{\text{Both zeros}\}} {\left(o_{ki}-o_{kj}\right)^{2}}\\ &\quad+ \sum_{k\in\{\text{One zero}\}}{\left(o_{ki}-o_{kj}\right)^{2}}. \end{aligned}  $$

For simplicity, we refer to all zeros in the gene expression data as dropout candidates. In general, our argument remains valid even when a dropout candidate is allowed to have near zero values. We note that the squared Euclidean distance in Eq. 1 can be arranged as a sum of three sum-of-squares terms. The first term is the sum of squared differences of *o*_*ki*_ and *o*_*kj*_ if they are both non-zero values. This term is not affected by dropouts. The second term is the sum of squared differences of *o*_*ki*_ and *o*_*kj*_ if they are both zeros, so this term is zero (or very small, if we include near zero values as dropout candidates).

Therefore, we observe that the main impact of dropouts comes from the third term, which deals with when one value is zero and the other is not. A zero can either represent a lack of gene expression in the ground truth or a dropout event in which a non-zero gene expression value is observed as a zero. If we treat all observed zeros as a lack of gene expression (therefore, treating the probability of a zero being a dropout event as zero), which is the case if we directly apply PCA to scRNA-seq data, this term will tend to be inflated. Nonetheless, it has been observed that the probability of a gene expression value being a dropout is inversely correlated with the true expression levels [1, 2]. This means a gene with low expression is more likely to become a dropout than a gene with high expression. Using this information, we hypothesize that we can shrink this dropout-induced inflation by imputing the expression value of a dropout candidate in the third term in Eq. 1 with its expected value given the dropout probability distribution. This is the motivation behind our new method CIDR (Clustering through Imputation and Dimensionality Reduction).

### The CIDR algorithm

The CIDR algorithm can be divided into the following five steps: (1) Identification of dropout candidates, (2) estimation of the relationship between dropout rate and gene expression levels, (3) calculation of dissimilarity between the imputed gene expression profiles for every pair of single cells, (4) PCoA using the CIDR dissimilarity matrix, and (5) clustering using the first few principal coordinates (Additional file 1: Figure S1).

CIDR first performs a logarithmic transformation on the tags per million (TPM) gene expression for each cell. The distribution of the log-transformed expression values in a scRNA-seq data set is typically characterized by a strong peak at zero, and one or more smaller non-zero positive peaks representing the expression of expressed genes [6, 15, 16].

For each cell *C*_*i*_, CIDR finds a sample-dependent threshold *T*_*i*_ that separates the zero peak from the rest of the expression distribution; Additional file 1: Figure S2a shows the distribution of tags for a library in a simulated data set. The red vertical line indicates the threshold *T*_*i*_. The entries for cell *C*_*i*_ with an expression of less than *T*_*i*_ are dropout candidates, and the entries with an expression of at least *T*_*i*_ are referred to as expressed. We call *T*_*i*_ the dropout candidate threshold. Note that dropout candidates include true dropouts as well as true low (or no) expressions.

The next step of CIDR involves estimating the relationship between dropout probability and gene expression levels. Let *u* be the unobserved true expression of a feature in a cell and let *P*(*u*) be the probability of it being a dropout. Empirical evidence suggests that *P*(*u*) is a decreasing function [1, 2]. CIDR uses non-linear least-squares regression to fit a decreasing logistic function to the data (empirical dropout rate versus average of expressed entries) as an estimate for *P*(*u*), illustrated by the tornado plot (Additional file 1: Figure S2b) for the simulated data set. By using the whole data set to estimate *P*(*u*), which we denote as $\hat {P}(u)$, we make the reasonable assumption that most dropout candidates in the data set are actually dropouts, and this allows the sharing of information between genes and cells.

$\hat {P}(u)$ is used for imputation in the calculation of the CIDR dissimilarity matrix. The dropout candidates are treated as missing values and we will now describe CIDR’s pairwise implicit imputation process. Consider a pair of cells *C*_*i*_ and *C*_*j*_, and their respective observed expressions *o*_*ki*_ and *o*_*kj*_ for a feature *F*_*k*_, and let *T*_*i*_ and *T*_*j*_ be dropout candidate thresholds defined as above. Imputation is applied only to dropout candidates, hence when *o*_*ki*_≥*T*_*i*_ and *o*_*kj*_≥*T*_*j*_ no imputation is required. Now consider the case in which one of the two expressions is below *T*_*i*_, say *o*_*ki*_<*T*_*i*_ and *o*_*kj*_≥*T*_*j*_. Then *o*_*ki*_ needs to be imputed and the imputed value $\hat {o}_{ki}$ is defined as the weighted mean

2 $$ \hat{o}_{ki} =\hat{P}\left(o_{kj}\right)o_{kj}+\left(1-\hat{P}(o_{kj})\right)o_{ki}.  $$

To achieve a fast speed in the implementation of the above step, we replace $\hat {P}(u)$ with a much simpler step function *W*(*u*), defined as

3 $$ W(u)=\left\{ \begin{array}{ll} 0, & \hat{P}(u)\leq T_{W}, \\ 1, & \hat{P}(u) > T_{W}, \end{array}\right.  $$

where *T*_*W*_ is by default 0.5. We refer to *W*(*u*) as the imputation weighting function, as it gives us the weights in the weighted mean in the imputation, and we refer to the jump of *W*(*u*), i.e., $\hat {P}^{-1}(T_{W})$, as the imputation weighting threshold (Additional file 1: Figure S2c). Therefore, the implemented version of Eq. 2 is

4 $$ \tilde{o}_{ki}= W\left(o_{kj}\right)o_{kj}+\left(1-W\left(o_{kj}\right)\right)o_{ki},  $$

where $\tilde {o}_{ki}$ is used as the imputed value of *o*_*ki*_. Lastly, if *o*_*ki*_<*T*_*i*_ and *o*_*kj*_<*T*_*j*_, we set both $\tilde {o}_{ki}$ and $\tilde {o}_{kj}$ to be zeros.

We have also implemented CIDR directly using $\hat {P}(u)$ without the step function simplification. As shown in Tables 1 and 3, the simplification step indeed speeds up the algorithm, and Tables 2 and 3 show that the step does not compromise clustering accuracy.

**Table 1:** Runtime comparison between CIDR and four other algorithms

Data set	Size	CIDR	CIDR (L)	prcomp	t-SNE	RaceID	ZIFA
Pancreatic islet	60	5.2 s	5.3 s	2.9 s	8.5 s	48.6 s	40.1 min
Simulation	150	1.9 s	2.3 s	2.9 s	14.2 s	20.7 s	32.1 min
Human brain	420	6.6 s	8.9 s	13.7 s	1.4 min	1.5 min	1.1 h
Mouse brain	1800	57.9 s	1.1 min	3.2 min	23.1 min	2.5 h^a^	1.8 h

CIDR is the default CIDR algorithm implementation with step function simplification, while CIDR (L) is the implementation with the non-simplified logistic function. The algorithms were run on a standard laptop: 2.8 GHz Intel Core i5 (I5-4308U), 8GB DDR3 RAM)

^a^RaceID failed to converge for the mouse brain data set

**Table 2:** Comparison of clustering accuracy (measured by adjusted rand index) between CIDR and four other algorithms

Data set	Size	CIDR	CIDR (L)	prcomp	t-SNE	RaceID	ZIFA
Pancreatic islet	60	0.68	0.42	0.21	0.20	0.22	0.20
Simulation	150	0.92	0.90	0.48	0.02	0	0.00
Human brain	420	0.90	0.88	0.48	0.57	0.39	0.53
Mouse brain	1800	0.52	0.37	0.26	0.62	0.37^a^	0.32

CIDR is the default CIDR algorithm implementation with step function simplification, while CIDR (L) is the implementation with the non-simplified logistic function

^a^RaceID failed to converge for the mouse brain data set

**Table 3:** Comparison of runtime and clustering accuracy (measured by adjusted rand index) between CIDR and four other algorithms on a simulation data set with 10,000 cells

Simulation (10K)	CIDR	CIDR (L)	prcomp	t-SNE	RaceID	ZIFA
Time	44.5 min	1.5 h	3.1 h	21.8 h	>14 day	1.6 day^a^
Adjusted rand index	0.99	1.00	0.99	0.00	N/A^b^	0.09

CIDR is the default CIDR algorithm implementation with step function simplification, while CIDR (L) is the implementation with the non-simplified logistic function. The algorithms except ZIFA were run on an AWS ec2 r3.2xlarge instance

^a^ZIFA ran out of memory on the AWS ec2 r3.2xlarge instance, and its runtime was recorded from a run on an AWS ec2 r3.8xlarge instance

^b^RaceID did not complete after 14 days

Then, the dissimilarity between *C*_*i*_ and *C*_*j*_ is calculated using Eq. 1 with the imputed values. We call this imputation approach implicit, as the imputed value of a particular observed expression of a cell changes each time it is paired with a different cell.

Dimensionality reduction is achieved by performing PCoA on the CIDR dissimilarity matrix. It is known that clustering performed on the reduced dimensions improves the results [17]. CIDR performs hierarchical clustering on the first few principal coordinates, and decides the number of clusters based on the Calinski–Harabasz index [18].

### Toy example

**Fig. 1:**
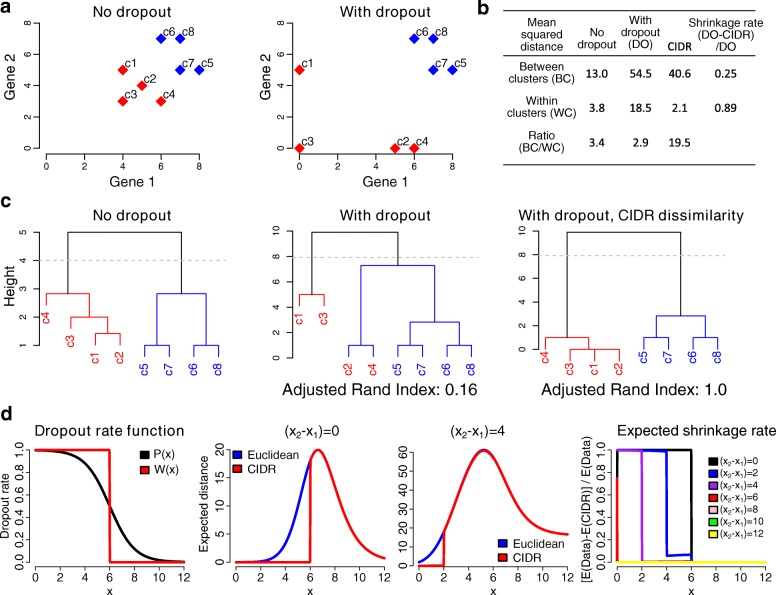
A toy example to illustrate the effect of dropouts in scRNA-seq data on clustering and how CIDR can alleviate the effect of dropouts. **a** This toy example consists of eight single cells divided into two clusters (the *red cluster* and the *blue cluster*). Dropout causes the within-cluster distances among the single cells in the *red cluster* to increase dramatically, as well as increasing the between-cluster distances between single cells in the two clusters. **b**CIDR reduces the dropout-induced within-cluster distances while largely maintaining the BC distances. **c** The hierarchical clustering results using the original data set (no dropout), the dropout-affected data set, and the dropout-affected data set analyzed using CIDR. *BC* between clusters, *DO* dropout, *scRNA-seq* single-cell RNA-seq, *WC* within clusters. **d** Using a step function W(x) to estimate the real dropout rate function P(x), we can show that *CIDR* always shrinks the expected distance between any two points (x_1_ and x_2_), and that the expected shrinkage rate is higher for those pairs of points that are closer together

Figure 1 shows a toy example that illustrates the effect of dropouts and how CIDR can improve clustering in the presence of dropouts. The toy data set consists of eight cells that form two clusters (the red cluster: c1–c4 and the blue cluster: c5–c8; Fig. 1 a). Dropouts affect mostly genes with lower expression levels, and hence has a greater impact on cells in the red cluster. Clustering quality can be quantified by the mean squared distance between every pair of cells within a cluster (WC distance) and between clusters (BC distance). The data set is said to have a strong clustering structure if it has low WC distances and high BC distances. In other words, a high ratio of BC/WC distances is an indication of good clustering structure. As illustrated in Fig. 1 a and b, dropouts increase both WC and BC distances. In this case, it also decreases the BC/WC ratio. Using the CIDR dissimilarity matrix, we were able to shrink greatly the mean WC distance, while mostly maintaining the mean BC distance. In other words, CIDR can shrink the WC distances more than the BC distances in a dropout-affected data set. As a result, CIDR is able to preserve better the clustering relationship in the original non-dropout data set (Fig. 1 c).

As a comparison, we have also considered an alternative method in which dropout candidates were imputed to the row mean (IRM) of the expressed entries. This is a straightforward and commonly used approach for dealing with data with missing values. When applying IRM to our toy data set, we observe that both the BC and WC distances shrink very significantly (Additional file 1: Figure S3). In fact, in this case IRM shrinks the BC distances much more than the WC distances, and therefore it dilutes the clustering signal.

This toy example illustrates that the power of CIDR comes from its ability to shrink dropout-induced WC distances while it largely maintain the BC distances. For a theoretical justification, see “Methods.”

### Simulation study

For an evaluation, we created a realistic simulated scRNA-seq data set. We set the number of markers for each cell type low to make it a difficult data set to analyze. Additional file 1: Figure S2a shows the distribution of tags for one randomly chosen library in this simulated data set. The spike on the left is typical for scRNA-seq data sets and the tags in this spike are dropout candidates. We compared CIDR with the standard PCA implemented by the R function prcomp, two state-of-the-art dimensionality-reduction algorithms (t-SNE and ZIFA), and the recently published scRNA-seq clustering package RaceID. As RaceID does not perform dimensionality reduction, the first two dimensions output by t-SNE were used in the two-dimensional visualization of RaceID. Since prcomp, ZIFA, and t-SNE do not perform clustering, for comparison, we applied the same hierarchical clustering procedure used by CIDR. We use the adjusted rand index [19] to measure the accuracy of clustering.

**Fig. 2:**
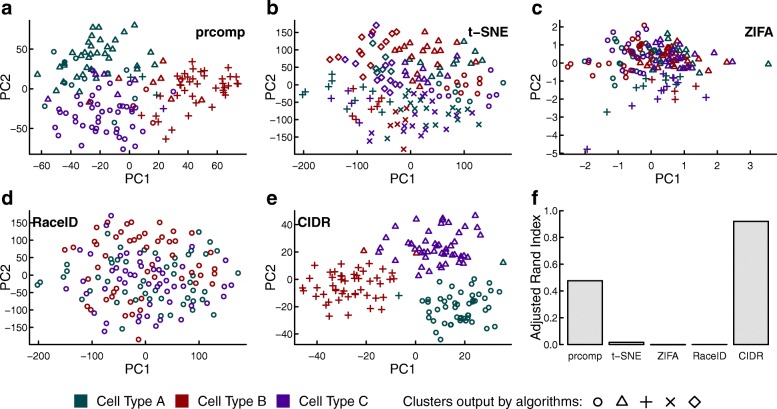
Performance evaluation with simulated data. Simulated scRNA-seq data set parameters: three cell types, 50 cells in each cell type, 20,000 non-differentially expressed features, 150 differentially expressed features and ten markers for each cell type. The three colors denote the three true cell types; while the different plotting symbols denote the clusters output by each algorithm. **a**–**e** Clustering output for each of the five compared algorithms. **f** The adjusted rand index is used to compare the accuracy of the clustering output for each of the compared algorithms. *PC* principal coordinates

As shown in Fig. 2, the only algorithm that displays three clearly recognizable clusters in the first two dimensions is CIDR. The accuracy of CIDR in cluster membership assignment is reflected by the adjusted rand index being much higher than those of the other four algorithms compared (Fig. 2 f). CIDR outputs all the principal coordinates as well as a plot showing the proportion of variation explained by each of the principal coordinates (Additional file 1: Figure S2d).

We perturbed the various parameters in the simulation study to test the robustness of CIDR and examine how its performance depends on these parameters. As expected, the adjusted rand index decreases as the dropout level or the number of cell types increases (Additional file 1: Figure S4a, c). However, when the adjusted rand index is low, the performance of CIDR can be improved to close to 1 by increasing the number of cells (Additional file 1: Figure S4b, d).

#### Scalability of CIDR

Given the ever increasing size of scRNA-seq data sets, and hence the importance of the speed of scRNA-seq data analysis software, we created a simulated data set of 10,000 cells to test the scalability of CIDR and the other algorithms. The results are shown in Table 3. CIDR completed the analysis within 45 min, which is more than four times faster than the second fastest algorithm prcomp (3.1 h), and many more times faster than t-SNE (21.8 h), ZIFA (1.6 days), or RaceID (which did not complete execution within 14 days). In fact, CIDR is the only algorithm that completed the analysis within an hour, while achieving a very high clustering accuracy (adjusted rand index =1).

### Biological data sets

We applied CIDR and the four compared algorithms on three very different biological data sets, for which the cell types are reported in the original publications. In these studies, cell types were determined through a multi-stage process involving additional information such as cell-type molecular signatures. For the evaluation and comparison, we applied each of the compared algorithms only once in an unsupervised manner to test how well each algorithm can recover the cell-type assignments in the studies.

#### Human brain scRNA-seq data set

**Fig. 3:**
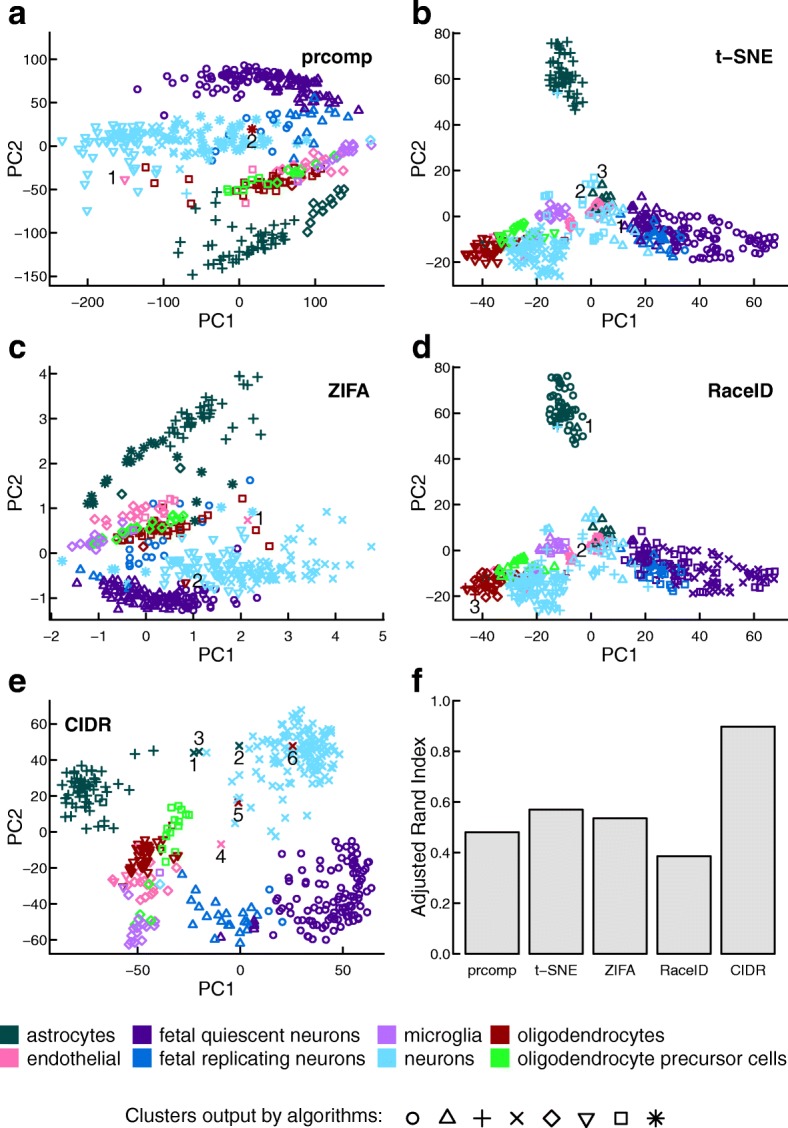
Performance evaluation with the human brain scRNA-seq data set. In this data set there are 420 cells in eight cell types after the exclusion of hybrid cells. The different colors denote the cell types annotated by the study [20], while the different plotting symbols denote the clusters output by each algorithm. **a**–**e** Clustering output for each of the five compared algorithms. **f** The adjusted rand index is used to measure the accuracy of the clustering output for each of the compared algorithms. Samples labeled by numbers are disagreements between the annotation and the clustering of the respective algorithm. *PC* principal coordinates

Figure 3 shows the comparison results for the human brain scRNA-seq data set [20]. In this data set, there are 420 cells in eight cell types after we exclude hybrid cells. Determining the number of clusters is known to be difficult in clustering; CIDR managed to identify seven clusters in the brain data set, which is very close to eight, the number of annotated cell types in this data set. CIDR also identified the members of each cell type largely correctly, as reflected by an adjusted rand index close to 0.9, which is a great improvement over the second best algorithm (Fig. 3 f). In the two-dimensional visualization by CIDR (Fig. 3 e), the first principal coordinate separates neurons from other cells, while the second principal coordinate separates adult and fetal neurons. Note that t-SNE is non-deterministic and it outputs dramatically different plots after repeated runs with the same input and the same parameters but with a different seed to the random number generator (Additional file 1: Figure S5).

CIDR allows the user to alter the number of principal coordinates used in clustering and the final number of clusters, specified by the parameters *nPC* and *nCluster* respectively. We altered these parameters and reran CIDR on the human brain scRNA-seq data set to test the robustness of CIDR (Additional file 1: Figure S6). When these parameters are altered from the default values, the clusters output by CIDR are still biologically relevant. For instance, 4 is recommended by CIDR as the optimal *nPC*, and in the resulting clustering, fetal quiescent neurons and fetal replicating neurons are output as two different clusters (Fig. 3 e); while when *nPC* is lowered to 2, these two types of cells are grouped as one cluster, i.e., fetal neurons (Additional file 1: Figure S6a).

**Fig. 4:**
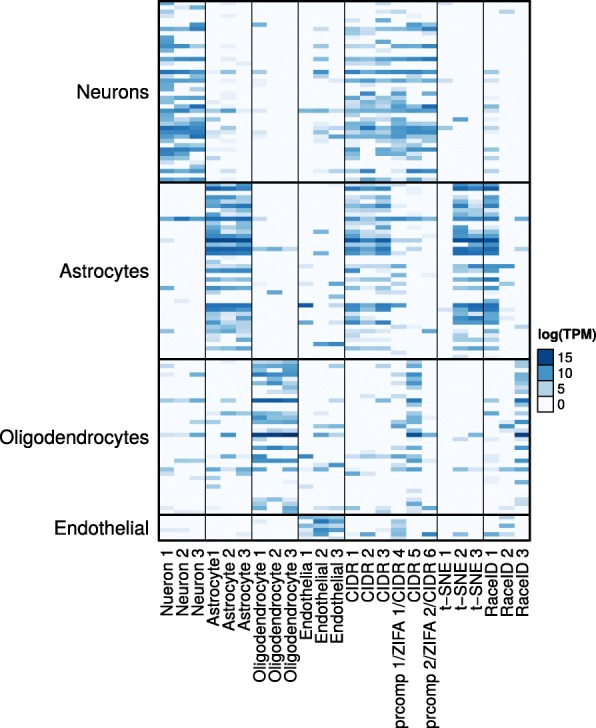
Expression of cell-type markers. Four groups of cell-type markers from an independent study [21]: neurons, astrocytes, oligodendrocytes, and endothelial cells. The *first* 12 *columns* are selected samples for which the annotation agrees with the CIDR clustering. *Columns* 13–18 are samples that are not annotated as neurons but clustered with neurons by CIDR, prcomp, or ZIFA. *Columns* 19–24 are selected samples that are not annotated as neurons but clustered with neurons by t-SNE or RaceID. *TPM* tags per million

We will now use the CIDR neuron cluster in the human brain scRNA-seq data set [20] as an example to illustrate how to use CIDR to discover limitations in the annotation. In Fig. 3 e, the cluster that corresponds best with the annotated neurons is denoted by crosses; there are only six disagreements, marked by 1–6 in Fig. 3 e, which are denoted by crosses but not annotated as neurons. We use cell-type markers from an independent study [21] to investigate the cause of these disagreements. In Fig. 4, these six samples are denoted by CIDR 1, CIDR 2, etc., and as all six samples express neuron markers, CIDR’s labels for them are justified. The first five out of these six samples express both neuron markers and the markers of the respective annotated cell types, suggesting that each of these samples contains RNAs from multiple cells, or they are potentially new cell types. The CIDR principal coordinates plot (Fig. 3 e) correctly places these five samples between neurons and the respective annotated cell types. The sixth sample expresses only neuron markers, suggesting a mistake in the annotation, and CIDR correctly places this sample in the middle of the neuron cluster. We carried out the same analysis using prcomp and ZIFA, and both methods can only identify CIDR 4 and CIDR 6, marked by 1 and 2, respectively, in Figs. 3 a and c. It is not possible to carry out this analysis using t-SNE or RaceID, because they incorrectly group neurons and other cell types in the same clusters. These errors are illustrated in Figs. 3 b, d, and 4, in which we can see that cells incorrectly grouped with neurons by t-SNE and RaceID, denoted by t-SNE 1, t-SNE 2, etc., have little expression in neuron markers.

#### Human pancreatic islet scRNA-seq data set

**Fig. 5:**
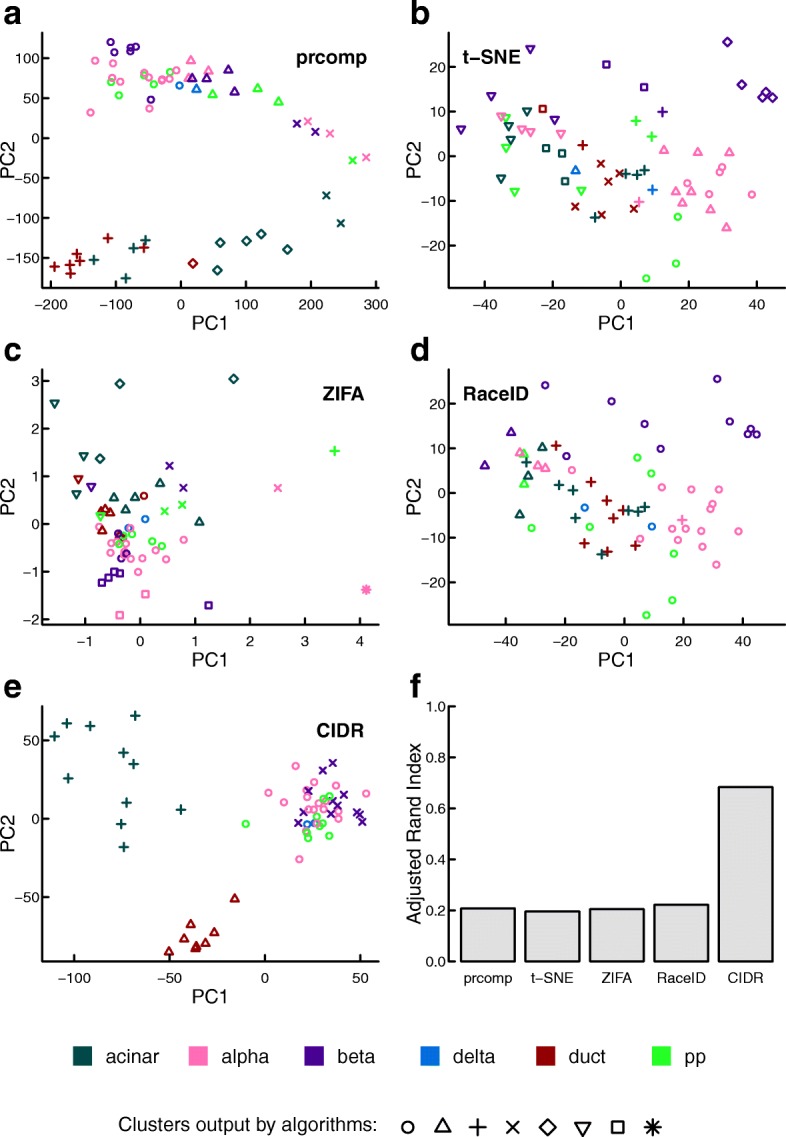
Performance evaluation on the human pancreatic islet scRNA-seq data set. In this data set, there are 60 cells in six cell types after the exclusion of undefined cells and bulk RNA-seq samples. The different colors denote the cell types annotated by the study [22], while the different plotting symbols denote the clusters output by each algorithm. **a**–**e** Clustering output for each of the five algorithms compared. **f** The adjusted rand index is used to measure the accuracy of the clustering output for each of the compared algorithms. *PC* principal coordinates

The human pancreatic islet scRNA-seq data set [22] has a smaller number of cells – 60 cells in six cell types – after we exclude undefined cells and bulk RNA-seq samples. CIDR is the only algorithm that displays clear and correct clusters in the first two dimensions (Fig. 5). Regarding clustering accuracy, CIDR outperforms the second best algorithm by more than threefold in terms of the adjusted rand index (Fig. 5 f).

#### Mouse brain scRNA-seq data set

In the mouse brain scRNA-seq data set [9], there are 1800 cells in seven cell types. Additional file 1: Figure S7 shows the results of the comparison using this data set. In this case, t-SNE achieves the highest adjusted rand index, and this is tightly followed by CIDR. Both t-SNE and CIDR perform much better than the other methods tested (Table 2 and Additional file 1: Figure S7), but CIDR (1 minute) is significantly faster than t-SNE (23 min) (Table 1). Also, we note that in the original publication [9], cell-type labels were assigned based on a multi-step procedure involving filtering and applying a modified bi-clustering algorithm, and the clustering results were visualized by t-SNE.

## Discussion and conclusion

CIDR has ultrafast runtimes, which are vital given the rapid growth in the size of scRNA-seq data sets. The runtime comparisons between CIDR and the other four algorithms over five data sets are shown in Tables 1 and 3. On a standard laptop, it takes CIDR only seconds to process a data set of hundreds of cells and minutes to process a data set of thousands of cells. *CIDR* is faster than prcomp and all the other compared algorithms; in particular, it is more than 50-fold faster than ZIFA, which is another dimensionality-reduction method that was specifically designed to deal with dropout in scRNA-seq data analysis.

Data preprocessing steps such as dimensionality reduction and clustering are important in scRNA-seq data analysis because detecting clusters can greatly benefit subsequent analyses. For example, clusters can be used as covariates in differential expression analysis [6], or co-expression analysis can be conducted within each of the clusters separately [23]. Certain normalization procedures should be performed within each of the clusters [5]. Therefore, the vast improvement CIDR has over existing tools will be of interest to both users and developers of scRNA-seq technology.

## Methods

### Dropout candidates

To determine the dropout candidate threshold that separates the first two modes in the distribution of tags (logTPM) of a library, CIDR finds the minimum point between the two modes in the density curve of the distribution. The R function density is used for kernel density estimation, and the Epanechnikov kernel is used as the smoothing kernel. For robustness, after calculating all the dropout candidate thresholds, the top and bottom 10 percentiles of the thresholds are assigned the 90th percentile and the 10th percentile threshold values, respectively. CIDR also gives the user the option of calculating the dropout candidate thresholds for only some of the libraries and in this option the median of the calculated thresholds is taken as the dropout candidate threshold for all the libraries.

In the kernel density estimation, CIDR uses the default bandwidth selection method nrd0 of the R function density with adjust = 1. We have varied the adjust parameter and re-calculated the adjusted rand indices for both the human brain [20] and human pancreatic [22] scRNA-seq data sets, and Additional file 1: Figure S8 shows that CIDR is robust with respect to this bandwidth adjustment. When the adjust parameter is varied from 0.5 to 1.5, the adjusted rand indices for CIDR for both the human brain and human pancreatic islet data sets stay much higher than the next best methods; see Figs. 3 f and 5 f.

### Dimensionality reduction

PCoA is performed on the CIDR dissimilarity matrix to achieve dimensionality reduction. Because the CIDR dissimilarity matrix does not, in general, satisfy the triangle inequality, the eigenvalues can possibly be negative. This does not matter as only the first few principal coordinates are used in both visualization and clustering, and their corresponding eigenvalues are positive. Negative eigenvalues are discarded in the calculation of the proportion of variation explained by each of the principal coordinates. Some clustering methods require the input dissimilarity matrix to satisfy the triangle inequality. To allow integration with these methods, CIDR gives the user the option of a Cailliez correction [24], implemented by the R package ade4. The corrected CIDR dissimilarity matrix does not have any negative eigenvalues.

### Determining the number of principal coordinates

CIDR implements an algorithm that is a variation of the scree [25] method for automatically determining the number of principal coordinates used in clustering. CIDR outputs a plot that shows the proportion of variation explained by each of the principal coordinates, and the scree approach looks for the elbow in the curve beyond which the curve flattens.

More specifically, CIDR assigns eigenvalues into groups based on the differences in consecutive eigenvalues. A new group is created each time a consecutive difference is greater than a cutoff point determined as a fraction of the largest difference. If the size of the current group exceeds a predetermined threshold, the sum of sizes of all but the current group is returned as the number of principal coordinates used in clustering.

Users are encouraged to inspect the proportion of variation plot output by CIDR, and possibly alter the number of principal coordinates used in clustering.

### Clustering

Hierarchical clustering is performed using the R package NbClust. CIDR’s default clustering method for hierarchical clustering is ward.D2 [26], and the number of clusters is decided according to the Calinski–Harabasz index [18]. The algorithm for cluster number decision is again a variation of the scree algorithm [25]. More specifically, the algorithm examines the second derivative of the Calinski–Harabasz index versus the number of clusters (Additional file 1: Figure S2e). Upon user request, CIDR can output the Calinski–Harabasz index versus the number of clusters plot; if needed, the user can alter the default number of clusters.

### Simulation study

Simulated log tags are generated from a log-normal distribution. For each cell type, an expected library, i.e., the true distribution of log tags, is first generated, and then dropouts and noise are simulated. For each cell type, the expected library includes a small number of differentially expressed features (e.g., genes and transcripts) and markers. By markers we mean features that are expressed in one cell type and are zeros in all other cell types.

A probability function *π*(*x*), where *x* is an entry in the expected library, is used to simulate dropouts. *π*(*x*) specifies how likely an entry is to be a dropout, so intuitively it should be a decreasing function. In our simulation, we use a decreasing logistic function. The parameters of the logistic function can be altered to adjust the level of dropouts. After simulating dropouts, Poisson noise is added to generate the final distribution for each library.

### Biological data sets

Tag tables from three recent scRNA-seq studies (human brain [20], human pancreatic islet [22], and mouse cerebral cortex [9]) were downloaded from the data repository NCBI Gene Expression Omnibus (GSE67835, GSE73727, and GSE60361). To ensure good quality, samples with a library size less than 10,000 were excluded. The raw tag tables were used as the inputs for CIDR. For the other dimensionality-reduction and clustering algorithms, rows with tag sums less than or equal to 10 were deleted. Log tags, with base 2 and prior count 1, were used as the inputs for ZIFA, as suggested by the ZIFA documentation. Data sets transformed by logTPM were used as inputs for prcomp and t-SNE.

### Theoretical justification

Here we show that CIDR always shrinks the expected distance between two dropout-affected samples (i.e., single cells), and has a higher expected shrinkage rate for WC distances than for BC distances. This property ensures that the CIDR dissimilarity matrix better preserves the clustering structure in the data set.

For simplicity of discussion, let us assume that dropouts are zeros. We will now explain why imputation by Eq. 2 in the main text improves clustering.

Suppose that a particular feature *F* has true expression levels *x*_1_, *x*_2_, and *x*_3_ for three cells *C*_1_, *C*_2_, and *C*_3_, respectively. Let us assume *x*_1_≤*x*_2_≤*x*_3_. Let *P* be the true dropout probability function, and $\hat {P}$ be the empirically estimated dropout probability function used in CIDR. Both *P* and $\hat {P}$ are monotonically decreasing functions, and satisfy $0\leq P,\hat {P}\leq 1$.

The true dissimilarity between *C*_1_ and *C*_2_ contributed by feature *F* is

$$D_{\text{true}}\left(C_{1}, C_{2}, F\right)=\left(x_{1}-x_{2}\right)^{2}. $$

In the presence of dropouts in the observed data, the expected value of dissimilarity between *C*_1_ and *C*_2_ contributed by feature *F* is

5 $$ {\begin{aligned} E\left(D_{\text{data}}\left(C_{1}, C_{2},F\right)\right)=& \left(1-P(x_{1})\right)\left(1-P(x_{2})\right)\left(x_{1}-x_{2}\right)^{2}\\ & +P(x_{2})\left(1-P(x_{1})\right)x_{1}^{2}\\ &+P(x_{1}) \left(1-P(x_{2})\right)x_{2}^{2}.\\ \end{aligned}}  $$

The expected value of the CIDR dissimilarity between *C*_1_ and *C*_2_ contributed by feature *F* is

6 $$ {\begin{aligned} E\left(D_{CIDR}\left(C_{1}, C_{2}, F\right)\right)=&\left(1-P\left(x_{1}\right)\right)\left(1-P(x_{2})\right)\left(x_{1}-x_{2}\right)^{2} \\ &+ P(x_{2})\left(1-P\left(x_{1}\right)\right)\left(1-\hat{P}(x_{1})\right)^{2} x_{1}^{2} \\ &+ P(x_{1})\left(1-P(x_{2})\right)\left(1-\hat{P}(x_{2})\right)^{2} x_{2}^{2}.\\ \end{aligned}}  $$

Comparing Eqs. 5 and 6, it is clear that the only difference is the presence of the factor $\big (1-\hat {P}(x_{i})\big)^{2}$ in the last two terms. Since $0\leq \hat {P}(x)\leq 1$, we can deduce that $\big (1-\hat {P}(x_{i})\big)^{2} \leq 1$, which means *E*(*D*_*CIDR*_(*C*_1_,*C*_2_,*F*))≤*E*(*D*_data_(*C*_1_,*C*_2_,*F*)) for the pair of cells *C*_1_ and *C*_2_. This demonstrates that CIDR shrinks the expected distance between two points in the presence of dropouts.

Furthermore, let us consider the expected rate of shrinkage between *C*_1_ and *C*_2_ contributed by feature *F*:

7 $$ {\begin{aligned} &E_{\text{shrinkage rate}}(C_{1}, C_{2}, F)\\ &\quad=\frac{E\left(D_{\text{data}}\left(C_{1}, C_{2}, F\right)\right)-E\left(D_{CIDR}\left(C_{1}, C_{2}, F\right)\right)}{E\left(D_{\text{data}}\left(C_{1}, C_{2}, F\right)\right)} \\ &\quad=1-\frac{E\left(D_{CIDR}\left(C_{1}, C_{2}, F\right)\right)}{E\left(D_{\text{data}}\left(C_{1}, C_{2}, F\right)\right)}. \end{aligned}}  $$

Let us consider *E*_shrinkage rate_(*C*_1_,*C*_2_,*F*) and *E*_shrinkage rate_ (*C*_1_,*C*_3_,*F*). Since CIDR always shrinks the expected distance between two points, and that $\big (1-\hat {P}(x_{3})\big)^{2} \geq \big (1-\hat {P}(x_{2})\big)^{2}$, our intuition is that *E*_shrinkage rate_(*C*_1_,*C*_3_,*F*) is likely smaller than or equal to *E*_shrinkage rate_(*C*_1_,*C*_2_,*F*). In other words, we hypothesize that the shrinkage rate between two closer points is larger than or equal to the shrinkage rate between two points that are further apart. It is very complex to prove this property algebraically, so we have conducted an extensive computational study on the rate of shrinkage. Additional file 1: Figure S9 shows that for a variety of monotonically decreasing *P* and $\hat {P}$, and for any fixed *x*_1_, the expected rate of shrinkage becomes smaller when *x*_2_ becomes larger. In particular, Additional file 1: Figure S9f shows the case when $\hat {P}$ is a step function. We observe that in all tested cases, our hypothesis holds. Therefore, we are satisfied that in practice CIDR shrinks WC distances more than BC distances due to this differential shrinkage rate property.

## Additional file

Additional file 1 Supplementary Figures S1–S9. CIDR: Ultrafast and accurate clustering through imputation for single-cell RNA-seq data (PDF 970 kb)

## References

[1] Pierson E, Yau C (2015). ZIFA: Dimensionality reduction for zero-inflated single-cell gene expression analysis. Genome Biol.

[2] Kharchenko PV, Silberstein L, Scadden DT (2014). Bayesian approach to single-cell differential expression analysis. Nat Methods.

[3] Scialdone A, Natarajan KN, Saraiva LR, Proserpio V, Teichmann SA, Stegle O (2015). Computational assignment of cell-cycle stage from single-cell transcriptome data. Methods.

[4] Buettner F, Natarajan KN, Casale FP, Proserpio V, Scialdone A, Theis FJ (2015). Computational analysis of cell-to-cell heterogeneity in single-cell RNA-sequencing data reveals hidden subpopulations of cells. Nat Biotechnol.

[5] Lun AT, Bach K, Marioni JC (2016). Pooling across cells to normalize single-cell RNA sequencing data with many zero counts. Genome Biol.

[6] Finak G, McDavid A, Yajima M, Deng J, Gersuk V, Shalek AK (2015). MAST: A flexible statistical framework for assessing transcriptional changes and characterizing heterogeneity in single-cell RNA sequencing data. Genome Biol.

[7] Trapnell C, Cacchiarelli D, Grimsby J, Pokharel P, Li S, Morse M (2014). The dynamics and regulators of cell fate decisions are revealed by pseudotemporal ordering of single cells. Nat Biotechnol.

[8] van der Maaten L, Hinton G (2008). Visualizing data using t-SNE. J Mach Learn Res.

[9] Zeisel A, Muñoz-Manchado AB, Codeluppi S, Lönnerberg P, La Manno G, Juréus A (2015). Cell types in the mouse cortex and hippocampus revealed by single-cell RNA-Seq. Science.

[10] Zurauskiene J, Yau C (2016). pcaReduce: Hierarchical clustering of single cell transcriptional profiles. BMC Bioinform.

[11] Kiselev VY, Kirschner K, Schaub MT, Andrews T, Yiu A, Chandra T, et al.SC3-consensus clustering of single-cell RNA-Seq data. bioRxiv. 2016:036558. 10.1038/nmeth.4236 PMC5410170 28346451

[12] Xu C, Su Z (2015). Identification of cell types from single-cell transcriptomes using a novel clustering method. Bioinformatics.

[13] Grün D, Lyubimova A, Kester L, Wiebrands K, Basak O, Sasaki N (2015). Single-cell messenger RNA sequencing reveals rare intestinal cell types. Nature.

[14] Prabhakaran S, Azizi E, Pe’er D. Dirichlet process mixture model for correcting technical variation in single-cell gene expression data. In: Proceedings of the 33rd International Conference on Machine Learning: 2016. p. 1070–9. PMC6004614 29928470

[15] McDavid A, Dennis L, Danaher P, Finak G, Krouse M, Wang A (2014). Modeling bi-modality improves characterization of cell cycle on gene expression in single cells. PLoS Comput Biol.

[16] Bacher R, Kendziorski C (2016). Design and computational analysis of single-cell RNA sequencing experiments. Genome Biol.

[17] Ronan T, Qi Z, Naegle KM (2016). Avoiding common pitfalls when clustering biological data. Sci Signal.

[18] Caliński T, Harabasz J (1974). A dendrite method for cluster analysis. Commun Stat.

[19] Hubert L, Arabie P (1985). Comparing partitions. J Classif.

[20] Darmanis S, Sloan SA, Zhang Y, Enge M, Caneda C, Shuer LM (2015). A survey of human brain transcriptome diversity at the single cell level. Proc Natl Acad Sci.

[21] Cahoy JD, Emery B, Kaushal A, Foo LC, Zamanian JL, Christopherson KS (2008). A transcriptome database for astrocytes, neurons, and oligodendrocytes: a new resource for understanding brain development and function. J Neurosci.

[22] Li J, Klughammer J, Farlik M, Penz T, Spittler A, Barbieux C (2016). Single-cell transcriptomes reveal characteristic features of human pancreatic islet cell types. EMBO Rep.

[23] Trapnell C (2015). Defining cell types and states with single-cell genomics. Genome Res.

[24] Cailliez F (1983). The analytical solution of the additive constant problem. Psychometrika.

[25] Cattell RB (1966). The scree test for the number of factors. Multivar Behav Res.

[26] Murtagh F, Legendre P (2014). Ward’s hierarchical agglomerative clustering method: which algorithms implement Ward’s criterion?. J Classif.

