# Supplementary material for: CIDR: Ultrafast and accurate clustering through imputation for single-cell RNA-seq data
Source: Genome Biol. 2017 Mar 28;18:59. doi: 10.1186/s13059-017-1188-0 (PMC5371246; doi:10.1186/s13059-017-1188-0)
Supplement: Additional file 1 — Supplementary Figures S1–S9. CIDR: Ultrafast and accurate clustering through imputation for single-cell RNA-seq data (PDF 970 kb) [file 13059_2017_1188_MOESM1_ESM.pdf]

Supplementary Figures

CIDR: Ultrafast and accurate clustering through imputation for single-cell RNA-seq data

Peijie Lin[1,2], Michael Troup[1] & Joshua W. K. Ho[1,2]

[1] *Victor Chang Cardiac Research Institute, Darlinghurst, NSW 2010, Australia.*

[2] *St Vincent's Clinical School, University of New South Wales, Darlinghurst, NSW 2010, Australia.*

Figure S1: *CIDR* **flowchart.** This flowchart illustrates the implicit imputation process through which *CIDR* calculates a dissimilarity matrix. The *CIDR* dissimilarity matrix is then used to perform three tasks: dimensionality reduction, data visualization and clustering.

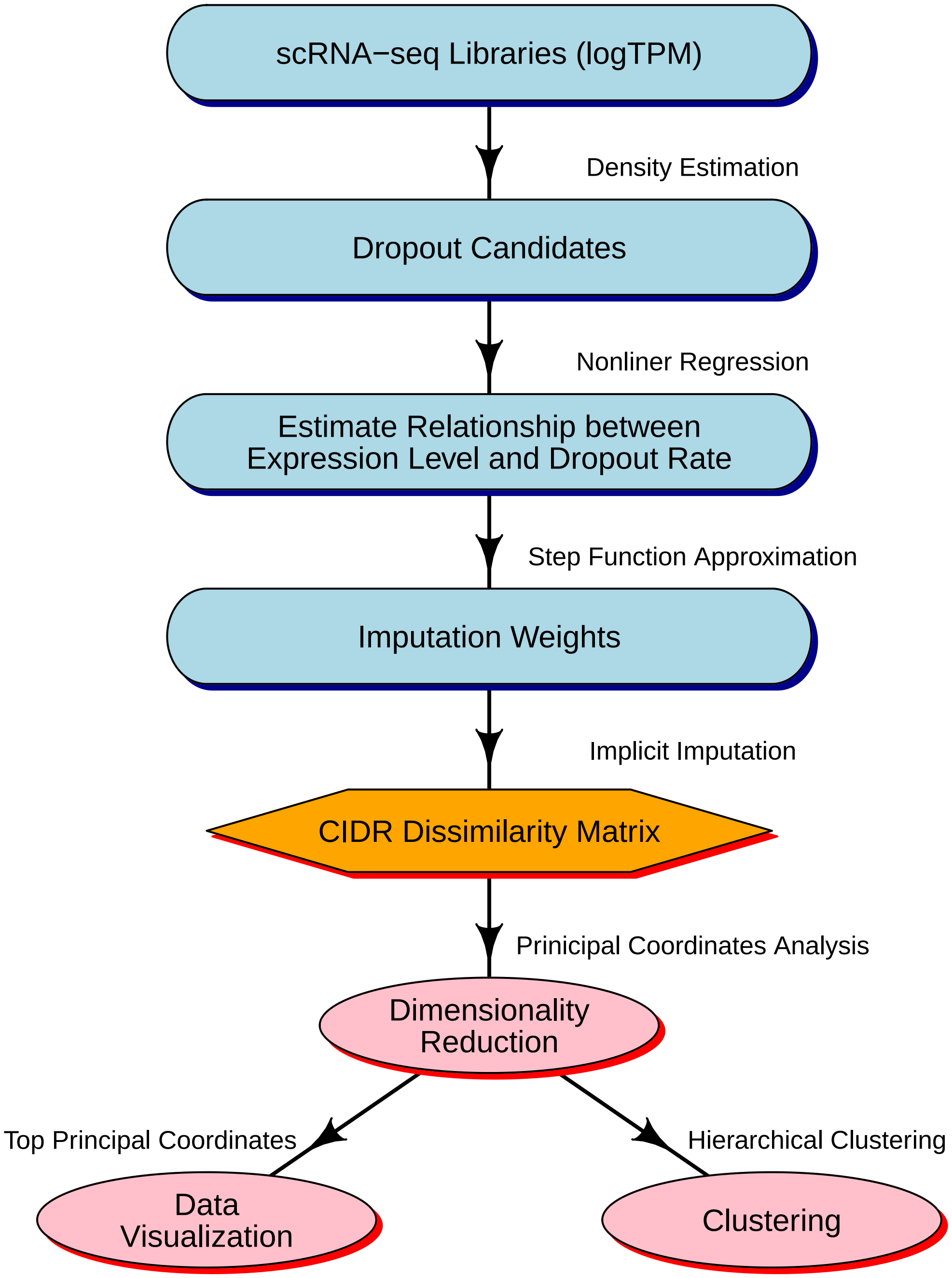

Figure S2: **Supplementary plots for the simulation study.** (a) Distribution of tags; (b) Tornado plot; (c) Imputation weighting function; (d) Proportion of variation explained by each of the principal coordinates; (e) Calinski-Harabasz Index versus Number of Clusters; (f) *CIDR* two-dimensional visualization.

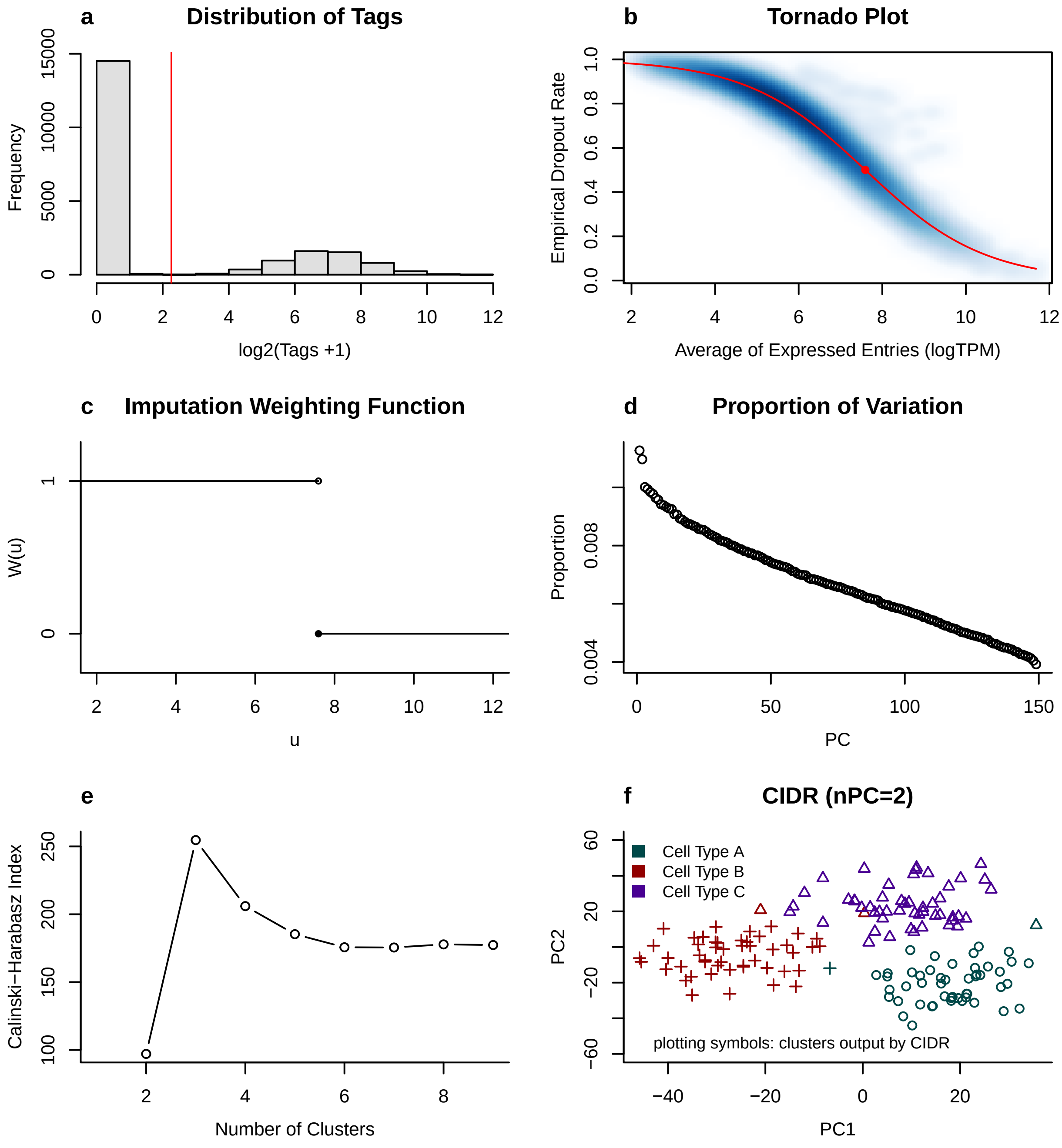

Figure S3: **Toy example: dropout candidates imputed to the row mean (IRM) of the expressed entries.** (a) Both the between- and within-cluster distances shrink very significantly. (b) IRM shrinks the between-cluster distances a lot more than the within-cluster distances, and therefore dilutes the clustering signal in the original non-dropout data set. (c) The hierarchical clustering result using IRM.

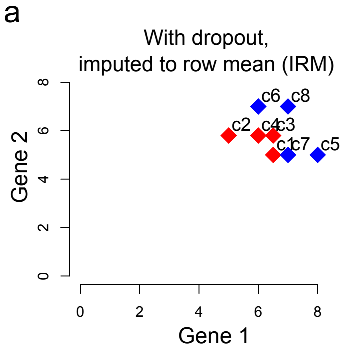

b

Mean squared distance	No dropout	With dropout (DO)	IRM	Shrinkage rate (DO-IRM) /DO
Between clusters (BC)	13.0	54.5	3.2	0.94
Within clusters (WC)	3.8	18.5	2.7	0.85
Ratio (BC/WC)	3.4	2.9	1.2	

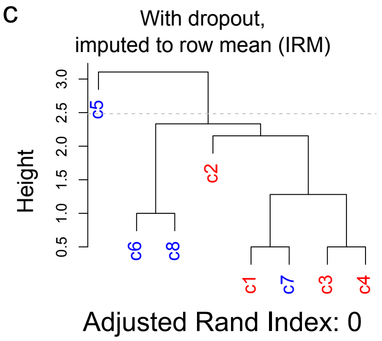

Figure S4: *CIDR* **performance on varying simulation parameters.** (a) Varying dropout level. A higher dropout level parameter means a higher level of dropouts. There are 3 cell types with 50 cells per cell type; (b) Varying number of cells in each cell type. There are 3 cell types, and the dropout level parameter is 9.6; (c) Varying number of cell types. There are 50 cells per cell type, and the dropout level parameter is 9.2; (d) Varying number of cells per cell type. There are 7 cell types, and the dropout level parameter is 9.2.

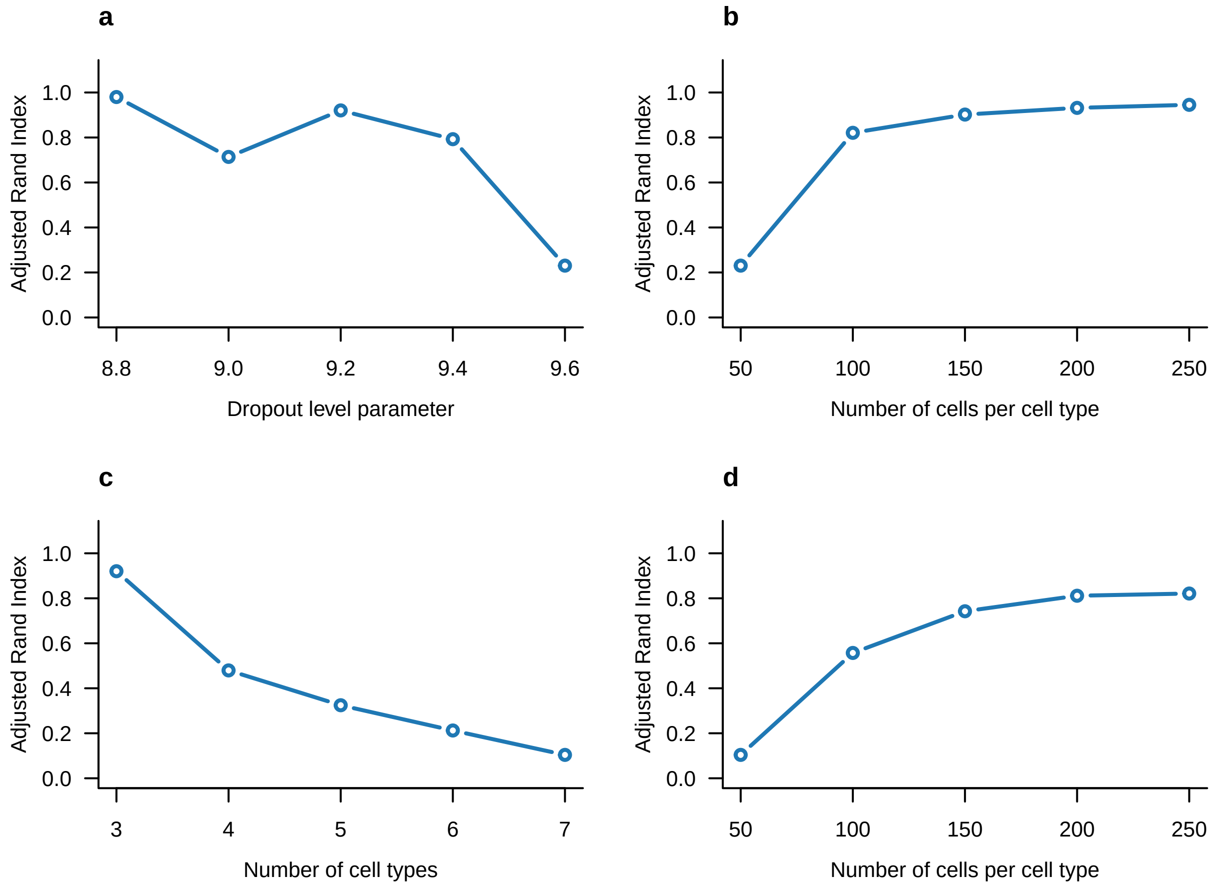

Figure S5: **Repeated *t-SNE* runs on the human brain scRNA-seq data set with the same parameters.** The different colors denote the cell types annotated by the study[1]. *t-SNE* is nondeterministic and it outputs dramatically different plots after repeated runs with the same input and the same parameters. The *t-SNE* parameters used here are $k = 4$ and $perplexity = 10$.

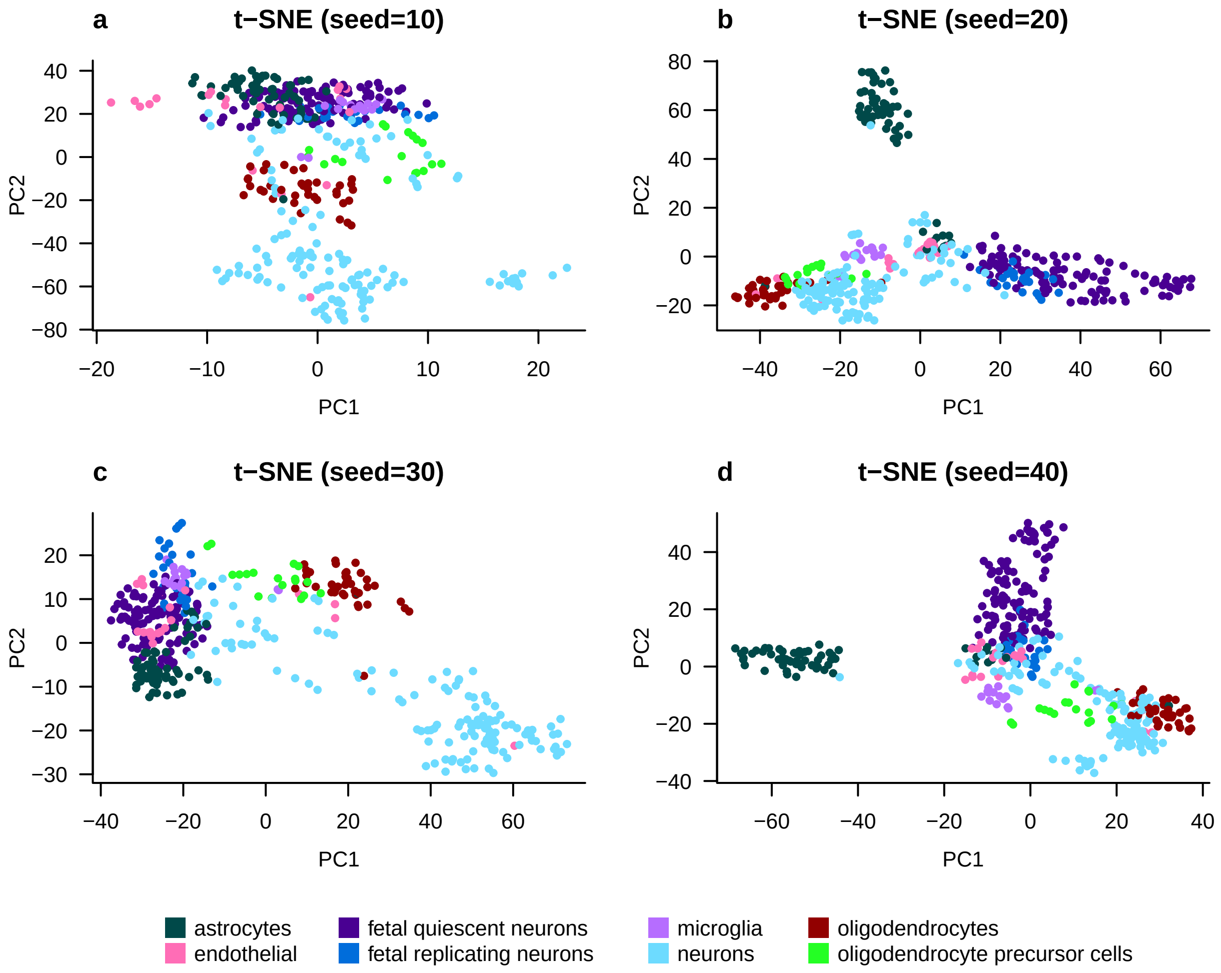

Figure S6: **Altering the parameters of *CIDR*.** The data set here is the human brain scRNA-seq data set[1]. (a) $nPC = 2$, $nCluster$ by default; (b) $nPC = 6$, $nCluster$ by default; (c) $nPC = 4$ (default), $nCluster = 5$; (d) $nPC = 4$ (default), $nCluster = 9$.

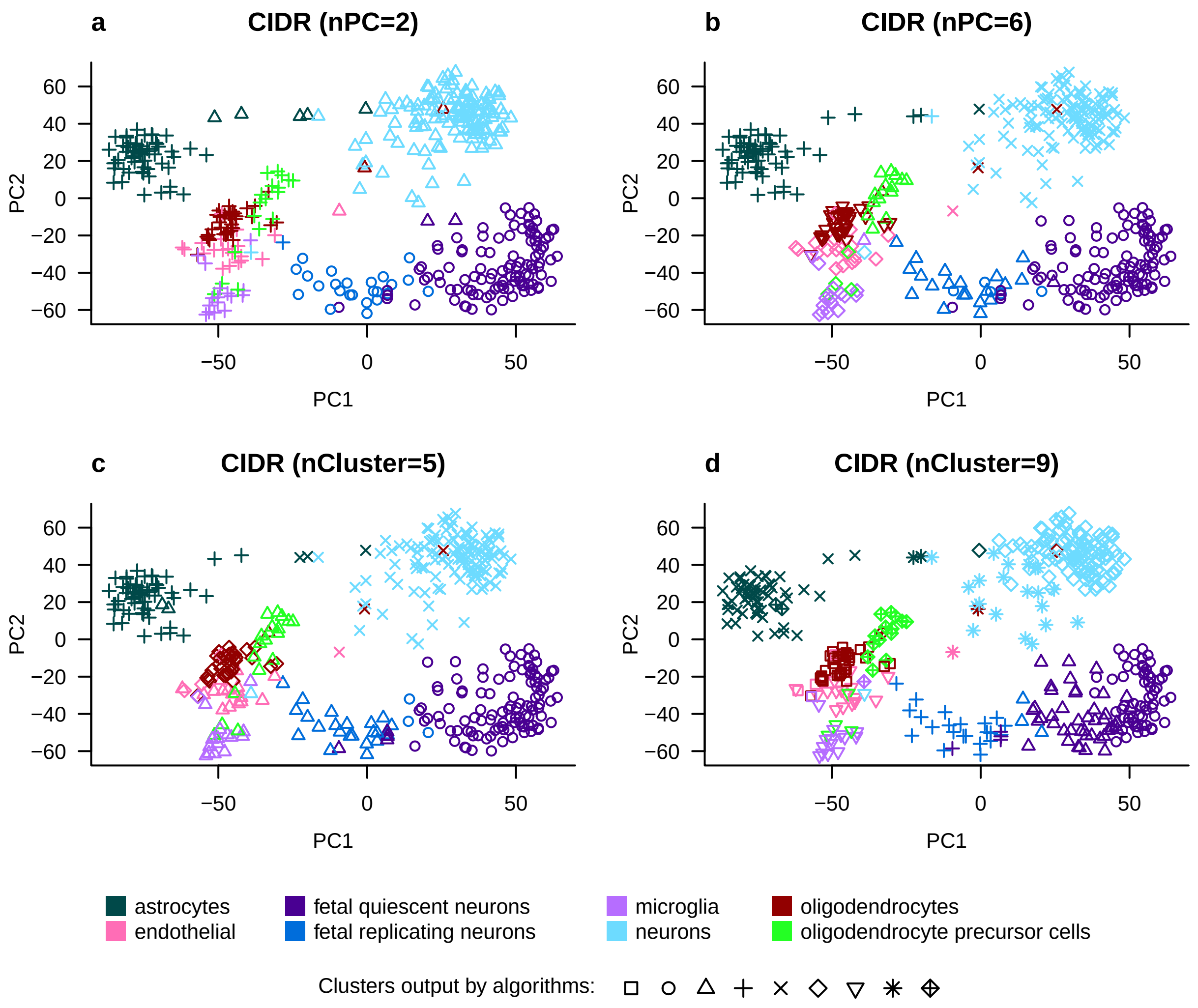

Figure S7: **Performance evaluation on the mouse brain scRNA-seq data set[2].** In this data set there are 1,800 cells in 7 cell types. The different colors denote the cell types annotated by the study; while the different plotting symbols denote the clusters output by each algorithm. (a) - (e) Clustering output by each of the five compared algorithms; (f) Adjusted Rand Index is used to measure the accuracy of the clustering output by each of the compared algorithms. Note that it is expected that *t-SNE* achieves a higher Adjusted Rand Index than *CIDR*, as the cell type annotation in the study was obtained through applying *t-SNE*.

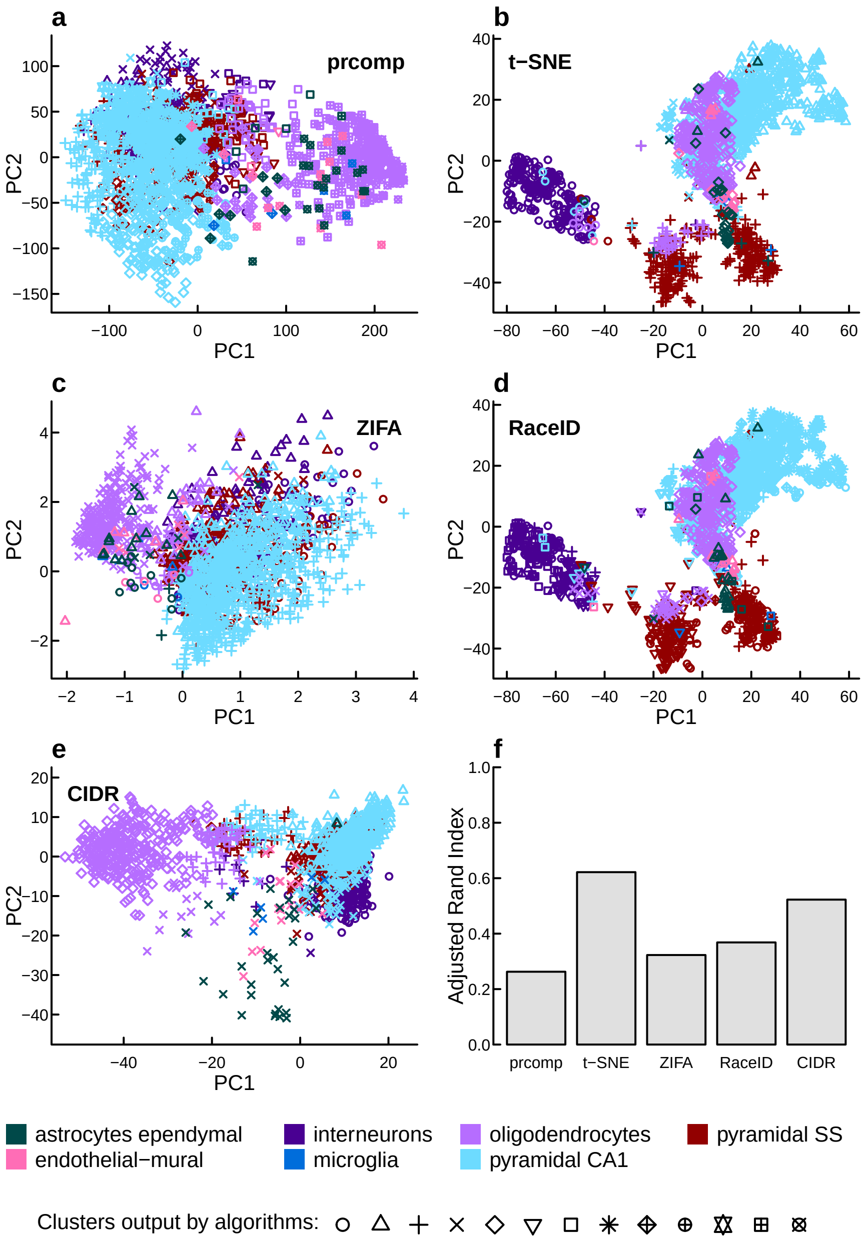

Figure S8: **Robustness of** *CIDR* **with respect to bandwidth adjustment.** *CIDR* uses the default *density* bandwidth selection method 'nrd0' with 'adjust = 1' in the kernel density estimation. Here we vary the 'adjust' parameter and re-calculate the Adjusted Rand Indices for both the human brain[1] and human pancreatic islet[3] scRNA-seq data sets.

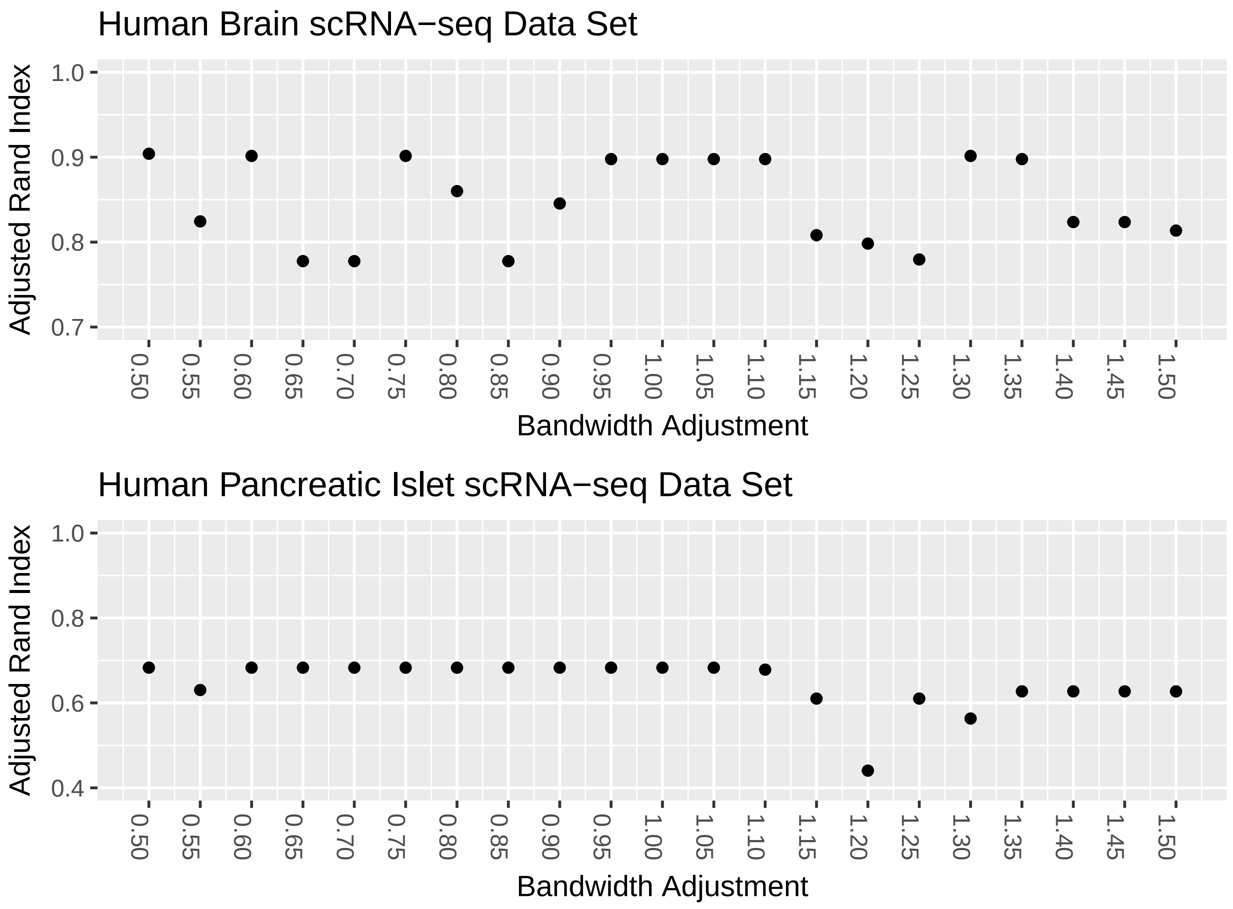

Figure S9: **Computational study on the rate of shrinkage.** For a variety of P and $\hat{P}$, and for any fixed x_1, the expected rate of shrinkage becomes smaller when x_2 becomes larger. This suggests that, on average, *CIDR* shrinks the distance between closer points more than it shrinks the distance between points that are further apart. This differential shrinkage property helps to reveal the underlying clustering structure in the original data.

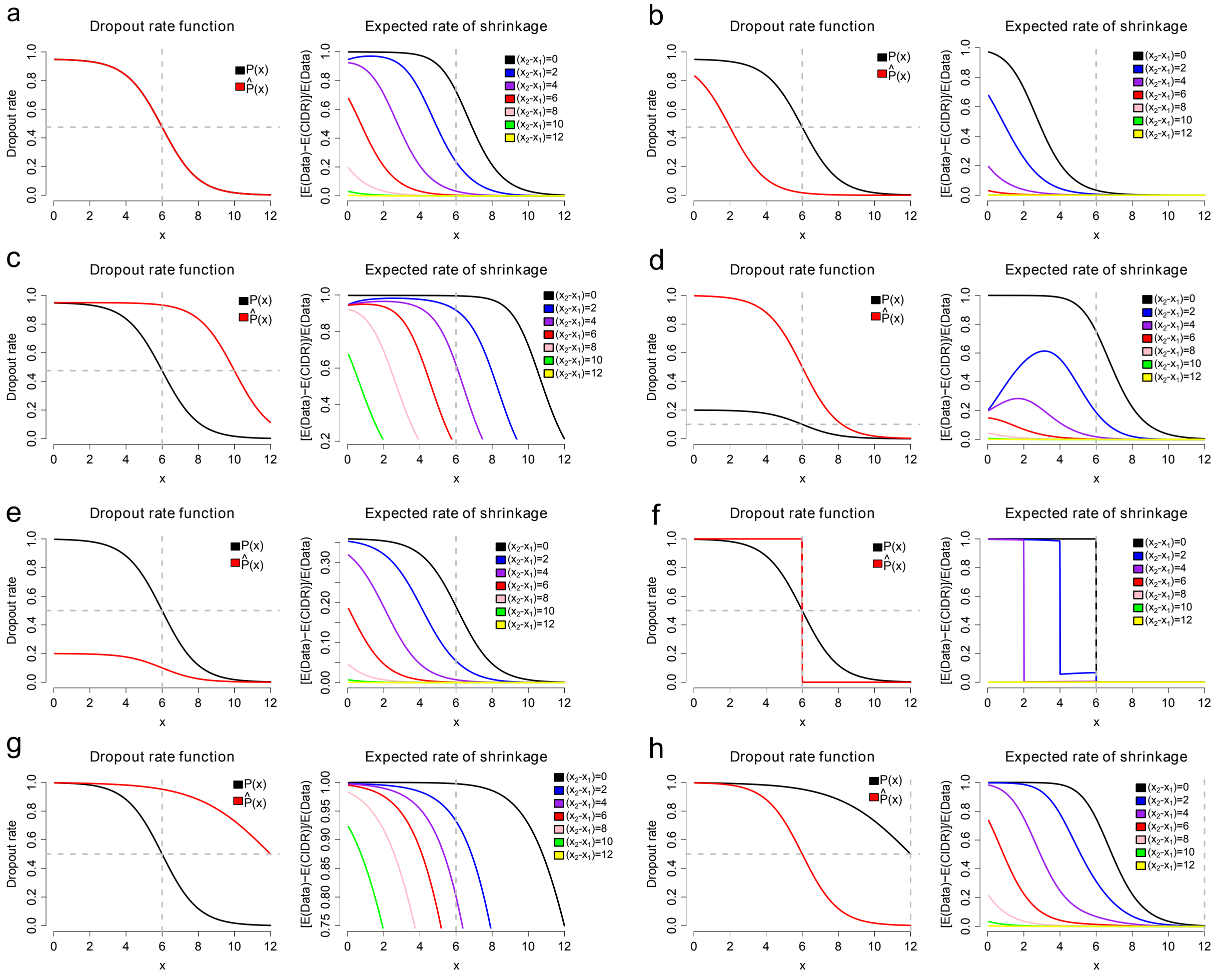

1. Darmanis, S., Sloan, S.A., Zhang, Y., Enge, M., Caneda, C., Shuer, L.M., Gephart, M.G.H., Barres, B.A., Quake, S.R.: A survey of human brain transcriptome diversity at the single cell level. Proceedings of the National Academy of Sciences **112**(23), 7285–7290 (2015)

2. Zeisel, A., Muñoz-Manchado, A.B., Codeluppi, S., Lönnerberg, P., La Manno, G., Juréus, A., Marques, S., Munguba, H., He, L., Betsholtz, C., *et al.*: Cell types in the mouse cortex and hippocampus revealed by single-cell RNA-seq. Science **347**(6226), 1138–1142 (2015)

3. Li, J., Klughammer, J., Farlik, M., Penz, T., Spittler, A., Barbieux, C., Berishvili, E., Bock, C., Kubicek, S.: Single-cell transcriptomes reveal characteristic features of human pancreatic islet cell types. EMBO Reports **17**(2), 178–187 (2016)
